# Mulberry Leaf Water Extract Ameliorates Impaired Glucose Tolerance via the Gut Microbiota/SCFAs/AMPKα1 Axis in Adipose Tissue

**DOI:** 10.3390/ph19081232

**Published:** 2026-08-05

**Authors:** Yang Yang, Yige Zhao, Yuhang Du, Jiamei Xie, Shuchang Liu, Gaiting Liu, Sitong Bu, Guohua Wang, Yongcheng An, Menglu Wang, Ziyi Shan, Baosheng Zhao

**Affiliations:** 1 Department of Pharmacology, School of Chinese Materia Medica, Beijing University of Chinese Medicine, Beijing 102488, China; bucmyang2020@163.com (Y.Y.); zyg408716172@163.com (Y.Z.); 20230935235@bucm.edu.cn (Y.D.); hbxiejiamei@163.com (J.X.); 15647039408@163.com (S.L.); gaiting2021@163.com (G.L.); colleen2003@163.com (S.B.); 17334054687@163.com (G.W.); 2 Department of Chemistry, Tsinghua University, Beijing 100084, China; hyss111@126.com; 3 Certification Center for Licensed Pharmacist of NMPA, Beijing 100061, China; wml1251247317@163.com; 4 Amway (China) Co., Ltd., Beijing 517399, China; 18645681606@163.com; 5 Beijing Research Institute of Chinese Medicine, Beijing University of Chinese Medicine, Beijing 100029, China

**Keywords:** impaired glucose tolerance, mulberry leaf water extract, adenosine 5′-monophosphate-activated protein kinase α1, gut microbiota, short-chain fatty acids, adipose-specific knockout mice

## Abstract

**Background/Objectives**: Impaired glucose tolerance (IGT) is a prediabetic condition affecting over 600 million adults worldwide. Mulberry leaf (*Morus alba* L.) has shown antidiabetic potential, but whether adipose AMPKα1 is required for its effects remains unclear. This study aimed to determine whether mulberry leaf water extract (MLE) ameliorates IGT through adipose AMPKα1-mediated gut microbiota modulation and thermogenesis. **Methods**: Adipose-specific AMPKα1 knockout (AKO) mice and floxed controls (Flox) were fed a high-fat diet for 12 weeks to establish IGT, then treated with MLE or short-chain fatty acids (SCFAs) for 10 weeks. Metabolic parameters, gut microbiota (16S rRNA sequencing), fecal SCFAs, and AMPKα1/SIRT1/PGC-1α pathway expression were assessed. **Results**: In Flox mice, MLE and SCFAs significantly improved glucose tolerance, reduced serum lipids and hepatic steatosis, and enhanced brown adipose tissue activation and white adipose browning. These effects were accompanied by gut microbiota remodeling, elevated fecal acetate/propionate/butyrate, and upregulation of the AMPKα1/SIRT1/PGC-1α pathway. All these metabolic and microbiota benefits were abrogated in AKO mice, in which MLE failed to improve any parameter, with only minimal and functionally insignificant thermogenic gene/protein upregulation. **Conclusions**: Adipose AMPKα1 is essential for MLE to improve IGT via gut microbiota/SCFAs and thermogenesis. These findings identify adipose AMPKα1 as a molecular target for dietary intervention in prediabetes.

## 1. Introduction

Impaired glucose tolerance (IGT) is clinically regarded as a prediabetic dysglycemic state, falling between normoglycemia and diabetic levels [1]. Without timely measures, IGT confers a substantial risk for the subsequent development of type 2 diabetes mellitus (T2DM) and its attendant cardiovascular sequelae [2]. IGT shares similar pathogenesis with T2DM, with insulin resistance resulting from reduced insulin sensitivity in skeletal muscle, liver, and adipose tissue being the core mechanism [3]. Current interventions for IGT primarily rely on lifestyle modifications and metformin therapy [4]. However, there is a growing demand for more effective and safer treatment strategies.

The gut microbiota exerts a pivotal influence on systemic metabolic pathways, as increasingly documented in recent research [5]. The anaerobic fermentation of non-digestible dietary carbohydrates by the gut microbiota yields a class of short-chain fatty acids (SCFAs), among which acetate, propionate, and butyrate are quantitatively dominant, comprising over 95% of the intestinal SCFA fraction [6]. SCFAs activate adenosine 5′-monophosphate-activated protein kinase (AMPK) [7], a key nutrient and energy sensor that maintains energy balance. Together, α, β, and γ subunits assemble into the heterotrimeric serine/threonine kinase AMPK [8]. Among them, the AMPKα1 isoform is widely expressed and particularly important in metabolic tissues. AMPK activation in adipose tissue simultaneously stimulates browning of white adipose tissue (WAT) and activates brown adipose tissue (BAT). These coordinated responses drive energy expenditure, facilitate glucose uptake, and promote thermogenesis [9].

Mulberry leaf (*Morus alba* L.) is a traditional Chinese herb commonly used to treat T2DM, first recorded in Shennong Bencao Jing (the earliest extant monograph on traditional Chinese medicine) for its property of “relieving Xiao Ke (consumptive-thirst disease, equivalent to diabetes in modern medicine)” [10]. Our previous studies revealed that mulberry leaf water extract (MLE) is enriched with polysaccharides, flavonoids, and alkaloids. Functionally, this extract improves glucose and lipid homeostasis in T2DM mice, an effect attributable to AMPK-mediated enhancement of both WAT browning and BAT activation [11]. In parallel, MLE restores gut microbial homeostasis and elevates SCFA levels in T2DM mice [12]. Nevertheless, how gut-adipose crosstalk contributes to mulberry leaf’s hypoglycemic action, particularly under IGT conditions, is not yet fully understood. Whether MLE acts through the gut microbiota/SCFAs/adipose AMPKα1 axis and whether AMPKα1 is an obligatory target requires further validation.

To address this scientific problem, we generated adipose tissue-specific AMPKα1 knockout (AKO) mice and performed parallel experiments with their floxed control (Flox) littermates. IGT model was established by feeding a 12-week high-fat diet (HFD), followed by a 10-week intervention with either MLE or SCFAs. Results showed that adipose-specific AMPKα1 knockout exacerbated HFD-induced IGT. MLE and SCFAs activated the AMPK/sirtuin 1 (SIRT1)/peroxisome proliferator-activated receptor γ coactivator 1α (PGC-1α)/uncoupling protein 1 (UCP1) axis to drive adipose thermogenesis, concomitant with significant improvements in glucose tolerance, remodeling of gut microbial ecology, enrichment of SCFA-producing taxa, and restoration of SCFA levels to normal ranges. Conversely, in the absence of adipose AMPKα1, the improvements in glucose tolerance, gut microbiota, and SCFAs were almost completely abrogated. Although thermogenic genes and proteins were still weakly upregulated, the magnitude was far lower than that observed in Flox controls. Collectively, this study demonstrates that adipose AMPKα1 is essential for mulberry leaf to ameliorate IGT, and that this action is mediated through the gut microbiota/SCFAs/adipose AMPKα1 cascade.

## 2. Results

### 2.1. Adipose-Specific AMPKα1 Deletion Exacerbated HFD-Induced IGT

**Figure 1:**
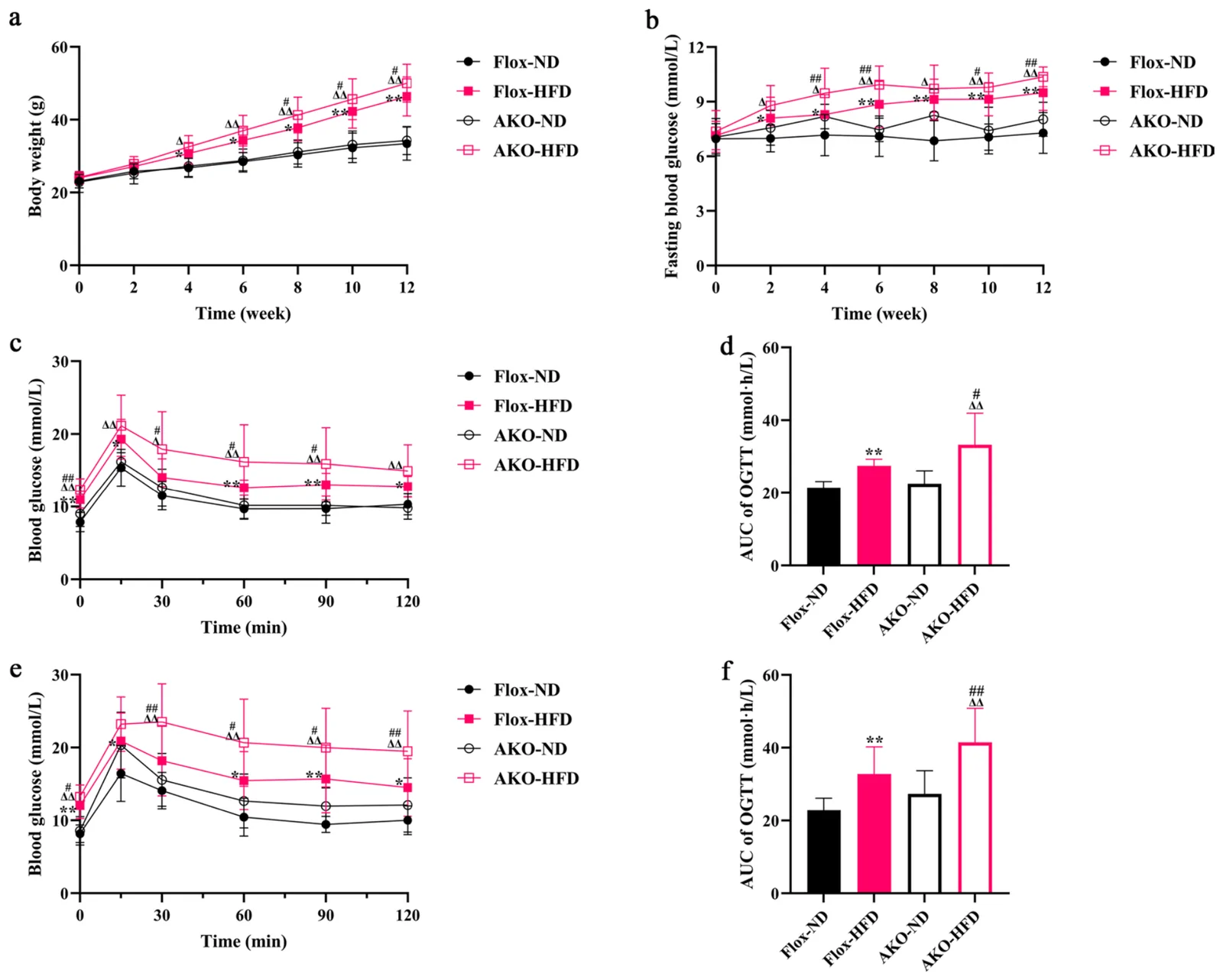
Adipose-specific AMPKα1 deletion exacerbated HFD-induced IGT. (**a**) Body weight. (**b**) Fasting blood glucose. (**c**) OGTT in week 10. (**d**) AUC of OGTT in week 10. (**e**) OGTT in week 12. (**f**) AUC of OGTT in week 12. Data are expressed as mean ± SD. *n* = 6 for the Flox-CD group, *n* = 18 for the Flox-HFD group, *n* = 6 for the AKO-CD group, and *n* = 18 for the AKO-HFD group. * *p* < 0.05, ** *p* < 0.01 vs. Flox-ND group. ^#^ *p* < 0.05, ^##^ *p* < 0.01 vs. Flox-HFD group. ^Δ^ *p* < 0.05, ^ΔΔ^ *p* < 0.01 vs. AKO-HFD group.

After 12 weeks of HFD, body weight, fasting glycemia, and oral glucose tolerance test (OGTT) were recorded to validate the IGT model. Body weight in HFD-fed mice was significantly higher than that in normal chow diet (ND)-fed mice starting from week 4 onward. From week 8, the AKO-HFD group had significantly higher body weight than the Flox-HFD group (Figure 1a). Fasting blood glucose levels progressively increased in the HFD groups, becoming significantly elevated relative to the ND groups from week 2. Notably, by week 12, blood glucose levels in the HFD groups remained below 11.1 mmol/L, indicating that the T2DM diagnostic threshold was not reached (Figure 1b). OGTT results at weeks 10 and 12 (Figure 1c,e) revealed severe glucose intolerance in the HFD groups compared to the ND groups, with the AKO-HFD group displaying more pronounced impairment than the Flox-HFD group, as shown by an increased area under the curve (AUC) in this group (Figure 1d,f). None of the parameters assessed differed significantly between the AKO-ND and Flox-ND groups. (Figure 1a–f). Collectively, the two consecutive OGTT results confirmed that the 12-week HFD feeding protocol reliably established a stable IGT model, and that adipose tissue-specific AMPKα1 deficiency exacerbates IGT under HFD conditions.

### 2.2. MLE and SCFAs Improved IGT and Metabolic Disorders Depend on AMPKα1 in Adipose Tissue

**Figure 2:**
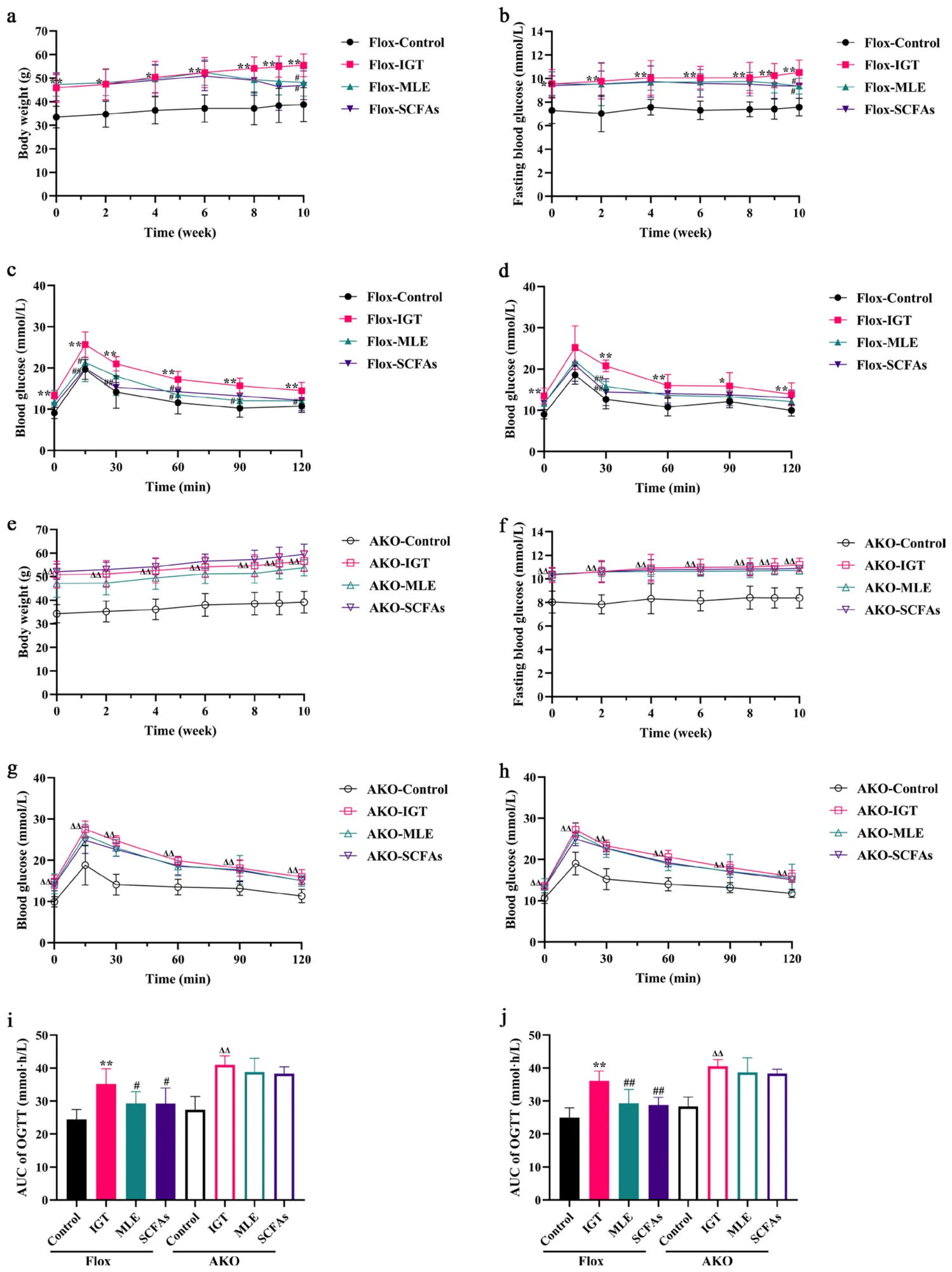
MLE and SCFAs alleviated HFD-induced body weight gain and ameliorated glucose intolerance in IGT mice through adipose tissue AMPKα1. For (**a**–**d**), Flox mice were treated for 10 weeks. (**a**) Body weight. (**b**) Fasting blood glucose. (**c**) OGTT in week 9. (**d**) OGTT in week 10. For (**e**–**h**), AKO mice were treated for 10 weeks. (**e**) Body weight. (**f**) Fasting blood glucose. (**g**) OGTT in week 9. (**h**) OGTT in week 10. (**i**) AUC of OGTT in week 9. (**j**) AUC of OGTT in week 10. Data are expressed as mean ± SD. *n* = 6. * *p* < 0.05, ** *p* < 0.01 vs. Flox-Control group. ^#^ *p* < 0.05, ^##^ *p* < 0.01 vs. Flox-IGT group. ^ΔΔ^ *p* < 0.01 vs. AKO-Control group.

After a 10-week intervention, in Flox mice, MLE and SCFAs administration significantly alleviated HFD-induced body weight gain and fasting hyperglycemia, and ameliorated glucose intolerance. Notably, body weight and fasting blood glucose levels in the Flox-MLE and Flox-SCFAs groups began to decline from the 8th week of treatment and became significantly lower than those in the Flox-IGT group by the 10th week (Figure 2a,b,e,f). OGTT results (Figure 2c,d) at weeks 9 and 10 revealed a marked improvement in glucose tolerance in both treatment groups, as reflected by a significant reduction in AUC compared with the Flox-IGT group (Figure 2i,j). In contrast, these beneficial effects were largely absent in AKO mice (Figure 2g–j).

**Figure 3:**
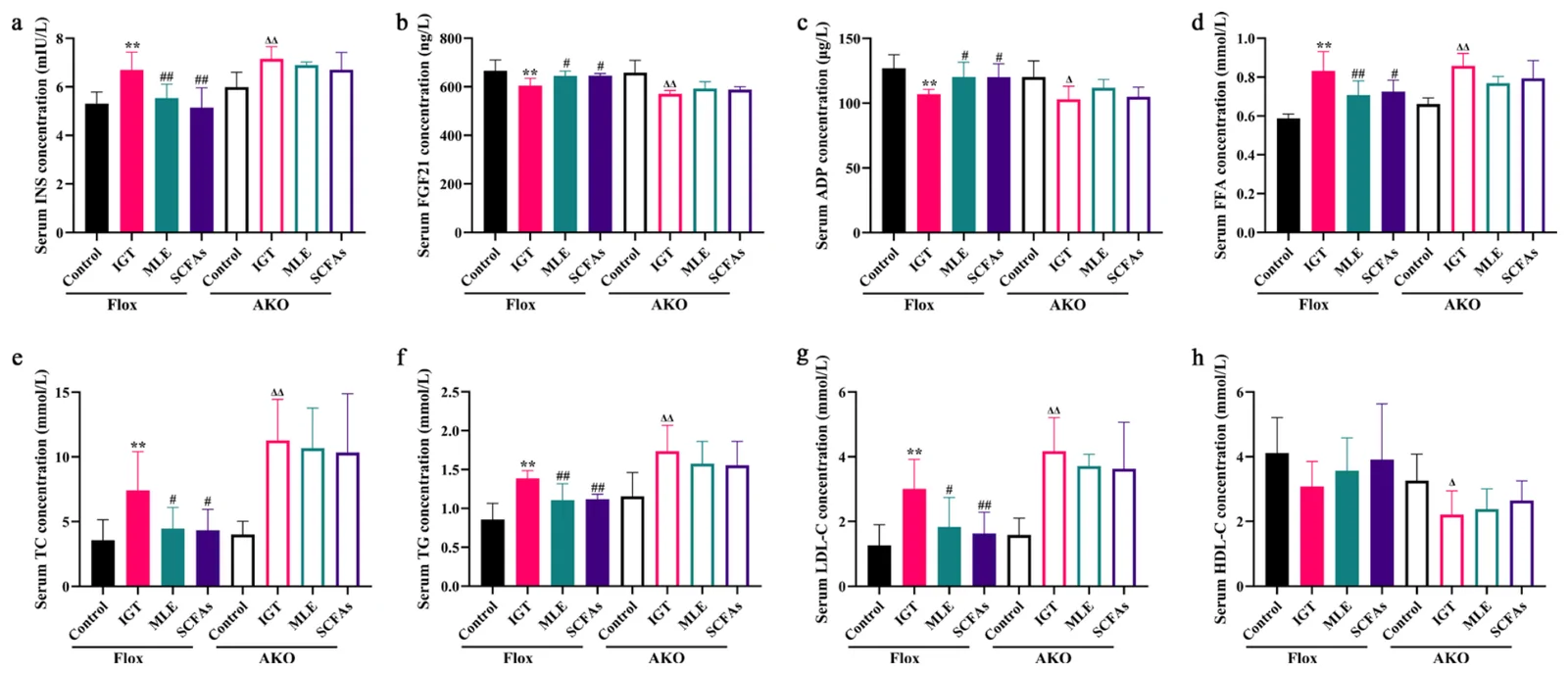
MLE and SCFAs ameliorated glucose and lipid metabolism disorders in IGT mice through adipose tissue AMPKα1. (**a**) Serum INS concentration. (**b**) Serum FGF21 concentration. (**c**) Serum ADP concentration. (**d**) Serum FFA concentration. (**e**) Serum TC concentration. (**f**) Serum TG concentration. (**g**) Serum LDL-C concentration. (**h**) Serum HDL-C concentration. Data are expressed as mean ± SD. *N* = 6, ** *p* < 0.01 vs. Flox-Control group. ^#^ *p* < 0.05, ^##^ *p* < 0.01 vs. Flox-IGT group. ^Δ^ *p* < 0.05, ^ΔΔ^ *p* < 0.01 vs. AKO-Control group.

Further assessment of glucose metabolism-related indicators showed that serum insulin (INS) levels were significantly elevated, while fibroblast growth factor 21 (FGF21) and adiponectin (ADP) levels were markedly reduced in the IGT groups (Figure 3a–c), indicating severe glucose metabolic dysregulation. After 10 weeks of treatment, the Flox-MLE and Flox-SCFAs groups exhibited significantly decreased serum INS and increased FGF21 and ADP levels (Figure 3a–c). However, no improvements were observed in AKO mice receiving the same treatments (Figure 3a–c).

Lipid metabolism parameters, including free fatty acids (FFA), total cholesterol (TC), triglycerides (TG), low-density lipoprotein cholesterol (LDL-C), and high-density lipoprotein cholesterol (HDL-C), were also evaluated. The IGT mice displayed profound dyslipidemia, characterized by markedly elevated serum FFA, TC, TG, and LDL-C levels, along with decreased HDL-C levels (Figure 3d–h). Notably, serum HDL-C in the AKO-IGT group was significantly lower than that in the AKO-Control group (Figure 3h), suggesting that adipose-specific AMPKα1 deletion exacerbates HFD-induced lipid disturbances. Following the 10-week intervention, the Flox-MLE and Flox-SCFAs groups showed substantial normalization of these abnormal lipid parameters, whereas no significant changes were detected in the AKO-MLE or AKO-SCFAs groups (Figure 3d–h).

Collectively, these results demonstrate that MLE and SCFAs effectively alleviate HFD-induced obesity, glucose intolerance, and dyslipidemia, and that these beneficial effects require the presence of AMPKα1 in adipose tissue.

### 2.3. MLE and SCFAs Alleviated Hepatic Steatosis and Systemic Inflammation via AMPKα1 Signaling

**Figure 4:**
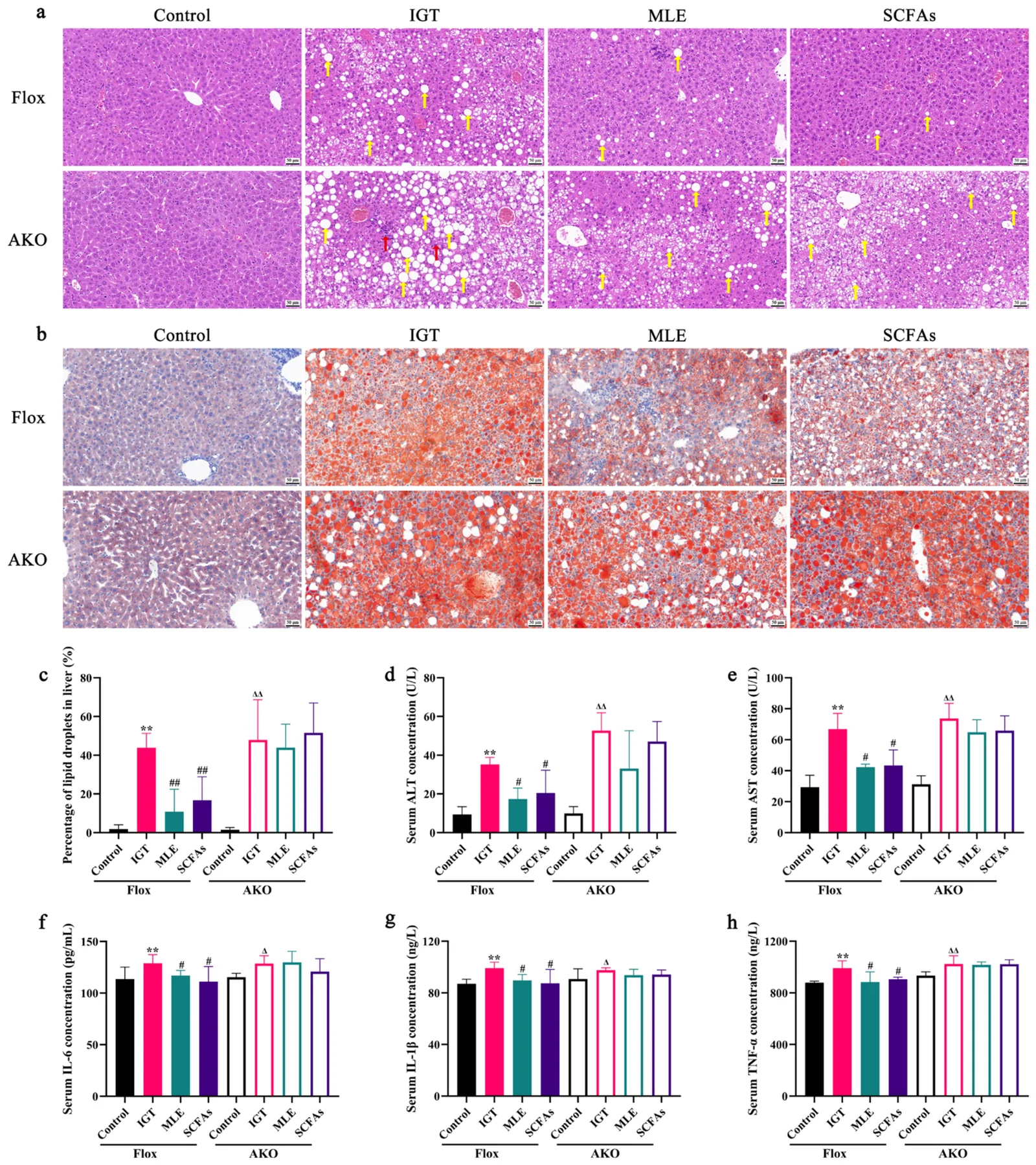
MLE and SCFAs improved hepatic lipid metabolism and inflammation in IGT mice via adipose tissue AMPKα1. (**a**) H&E staining of liver tissue (×20, scale bar = 50 μm; *n* = 3). Red arrows indicate inflammatory cell infiltration. Yellow arrows indicate hepatocyte vacuolation (steatosis). (**b**) Oil red O staining of liver tissue (×20, scale bar = 50 μm; *n* = 3). Red staining represents lipid droplets. (**c**) Percentage of lipid droplets in liver (*n* = 3). (**d**) Serum ALT concentration (*n* = 6). (**e**) Serum AST concentration (*n* = 6). (**f**) Serum IL-6 concentration (*n* = 6). (**g**) Serum IL-1β concentration (*n* = 6). (**h**) Serum TNF-α concentration (*n* = 6). Data are expressed as mean ± SD. ** *p* < 0.01 vs. Flox-Control group. ^#^ *p* < 0.05, ^##^ *p* < 0.01 vs. Flox-IGT group. ^Δ^ *p* < 0.05, ^ΔΔ^ *p* < 0.01 vs. AKO-Control group.

Previous studies have indicated that deficiency of AMPKβ1/β2 subunits in adipocytes predisposes to hepatic lipid accumulation and insulin resistance [13]. Therefore, we further investigated the impact of adipocyte-specific AMPKα1 deletion on liver metabolism in the two genotypes. Hematoxylin and eosin (H&E) and Oil Red O staining were performed to assess hepatic morphology and lipid droplet content. Under normal diet conditions, the presence or absence of adipose AMPKα1 did not noticeably affect liver histology (Figure 4a–c). However, under HFD feeding that induced glucose intolerance, IGT mice exhibited hepatocyte swelling, steatosis, and substantial lipid deposition (Figure 4a–c). Notably, the AKO-IGT group additionally displayed inflammatory cell infiltration (Figure 4a). Consistent with these histological changes, serum concentrations of aspartate aminotransferase (AST) and alanine aminotransferase (ALT) were markedly elevated, and the levels of pro-inflammatory cytokines interleukin-6 (IL-6), interleukin-1β (IL-1β), and tumor necrosis factor-α (TNF-α) were significantly increased in IGT mice (Figure 4d–h), indicating severe hepatotoxicity and excessive inflammatory responses.

Following 10 weeks of treatment with either MLE or SCFAs, the Flox mice showed restored liver architecture and a significant decrease in lipid droplet accumulation (Figure 4a–h). In contrast, no significant improvement was observed in AKO mice receiving the same treatments (Figure 4a–h). These results confirm that the ameliorative effects of MLE and SCFAs on hepatic metabolism and inflammation in IGT mice require AMPKα1.

### 2.4. MLE and SCFAs Enhanced Adaptive Thermogenesis by Increasing BAT Activity via AMPKα1

**Figure 5:**
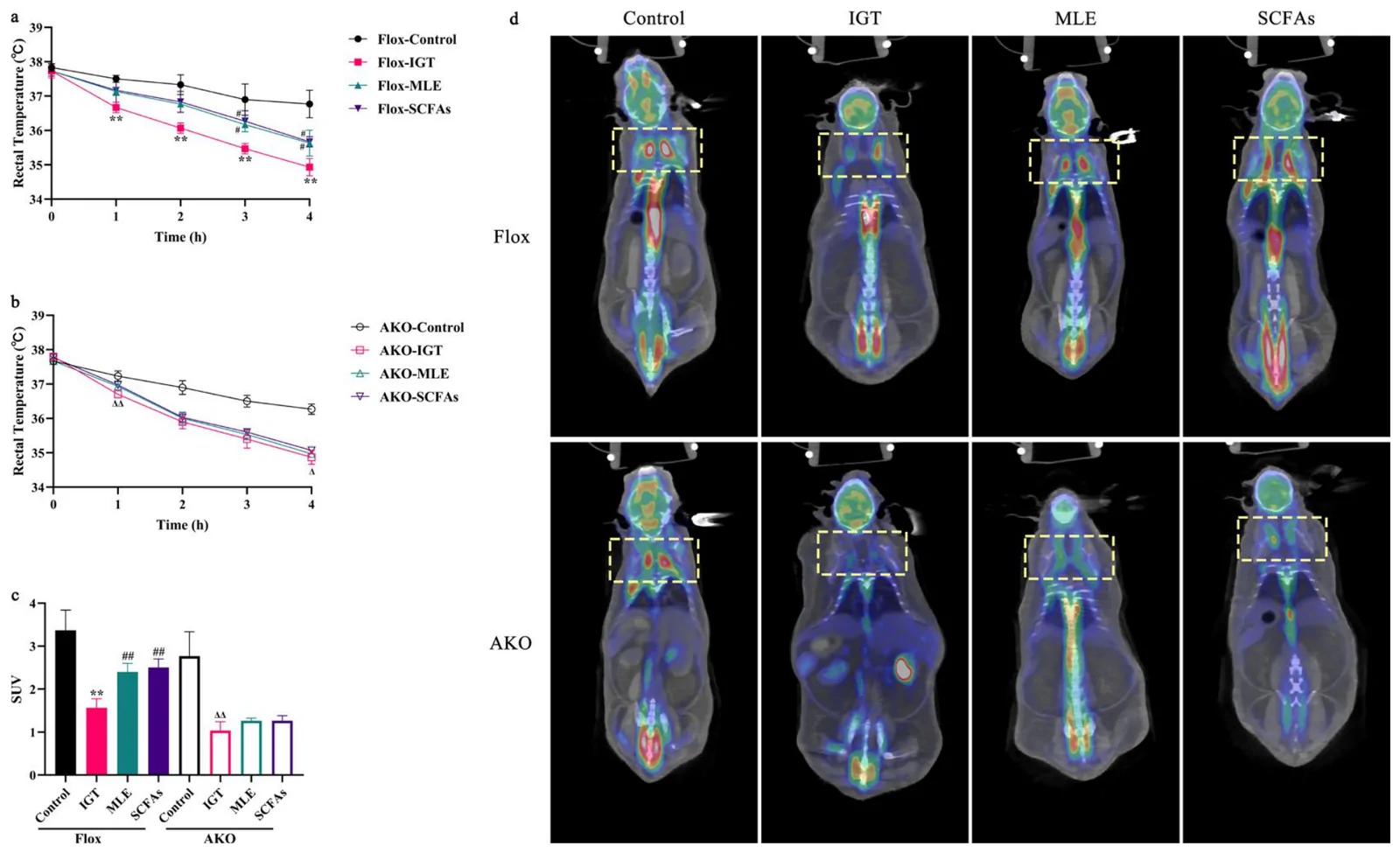
MLE and SCFAs increased adaptive thermogenesis and BAT activity in IGT mice via AMPKα1. (**a**) Cold tolerance test in Flox mice. (**b**) Cold tolerance test in AKO mice. (**c**) SUV of [^18^F]-FDG in BAT. (**d**) Micro-PET/CT imaging of BAT. The yellow dashed box indicates the interscapular BAT region. The color gradient from blue to red represents the SUV of [^18^F]-FDG, reflecting BAT metabolic activity. Data are expressed as mean ± SD. *n* = 3. ** *p* < 0.01 vs. Flox-Control group. ^#^ *p* < 0.05, ^##^ *p* < 0.01 vs. Flox-IGT group. ^Δ^ *p* < 0.05, ^ΔΔ^ *p* < 0.01 vs. AKO-Control group.

Adipose tissue serves a key function in thermogenesis and body temperature maintenance. After 10 weeks of treatment, mice were placed at 4 °C and monitored for thermoregulatory performance during cold exposure to assess cold tolerance. IGT mice showed a sharp drop in core temperature upon cold exposure (Figure 5a,b). In Flox mice, administration of MLE or SCFAs significantly attenuated the cold-induced drop in body temperature (Figure 5a). In contrast, AKO mice receiving the same interventions still showed a rapid temperature decrease (Figure 5b), indicating impaired cold adaptation.

BAT activity is closely linked to thermogenesis [14]. In our experiment, BAT function was assessed using standardized [^18^F]-FDG uptake measurements. PET imaging documented markedly attenuated BAT activity in IGT mice relative to their respective controls (Figure 5d). After MLE or SCFAs treatment, Flox mice showed increased [^18^F]-FDG utilization in BAT, whereas only a weak signal was detected in the AKO treatment groups (Figure 5d). Quantitative analysis of BAT was performed using Inveon Research Workplace software to calculate standardized uptake value (SUV). Consistent with the imaging findings, SUV elevation in the Flox-MLE and Flox-SCFAs groups was significantly greater than that in the Flox-IGT group (Figure 5c). Nevertheless, SUV values in the AKO-MLE and AKO-SCFAs groups did not differ significantly from those in the AKO-IGT group (Figure 5c).

Collectively, these results demonstrate that MLE and SCFAs drive BAT activation, thereby facilitating adaptive thermogenesis in an AMPKα1-dependent manner.

### 2.5. MLE and SCFAs Promoted iWAT Browning and BAT Activation, Requiring AMPKα1

**Figure 6:**
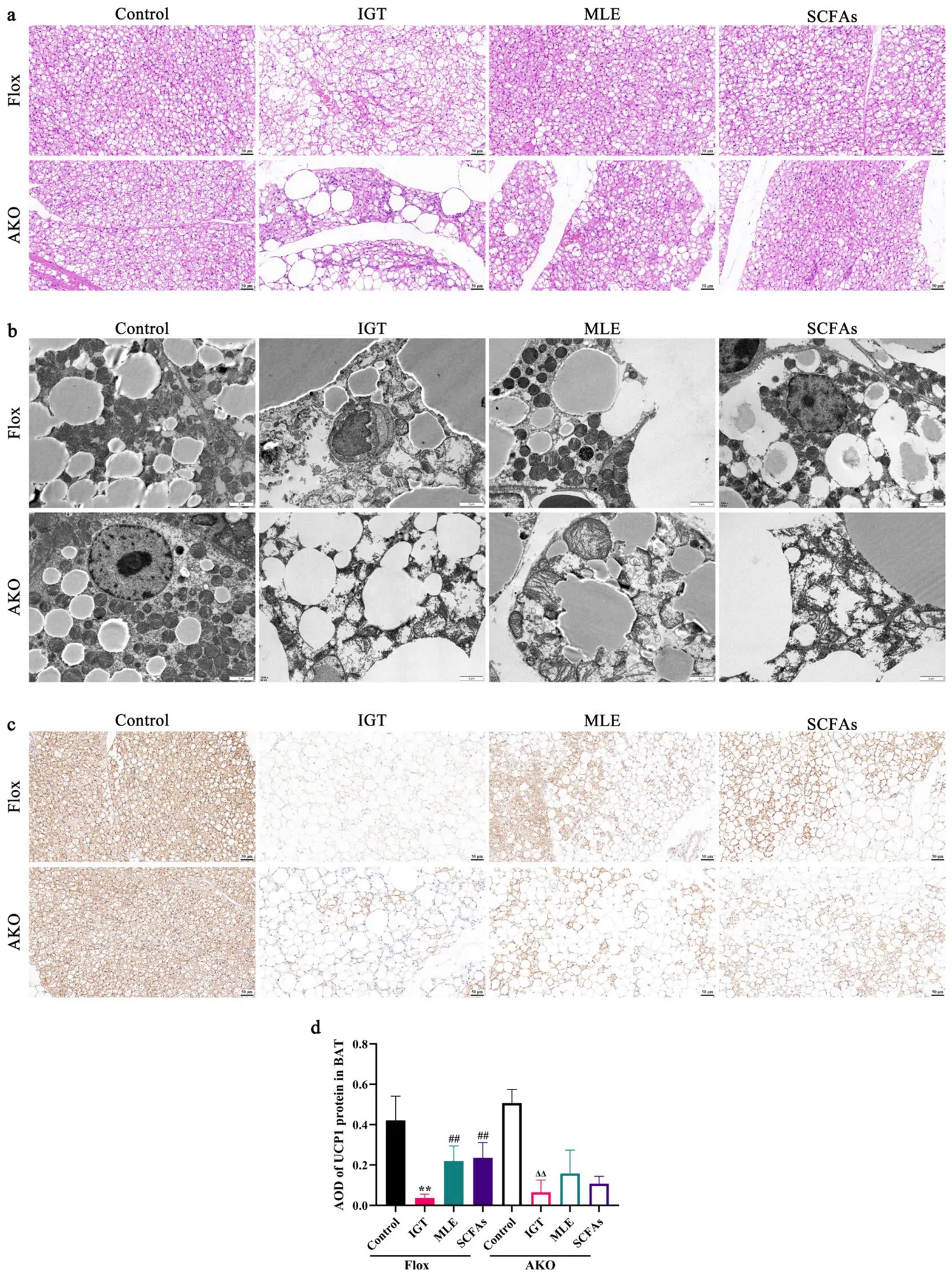
MLE and SCFAs improved BAT architecture and promoted BAT activation in IGT mice via AMPKα1. (**a**) H&E staining of BAT (×20, scale bar = 50 μm). (**b**) Immunohistochemical staining of UCP1 in BAT (×20, scale bar = 50 μm). (**c**) BAT ultrastructure examined by TEM (×2500, scale bar = 2 μm). (**d**) Quantitative BAT UCP1 protein expression. Data are expressed as mean ± SD. *n* = 3. ** *p* < 0.01 vs. Flox-Control group. ^##^ *p* < 0.01 vs. Flox-IGT group. ^ΔΔ^ *p* < 0.01 vs. AKO-Control group.

Our findings indicate that MLE and SCFAs stimulate both BAT activity and thermogenesis. A hallmark of BAT activation is the presence of multilocular adipocytes, along with enhanced mitochondrial content and elevated UCP1 expression [15]. Histological assessment by H&E staining showed that BAT from Flox-MLE and Flox-SCFAs groups contained markedly smaller and more numerous lipid droplets than the Flox-IGT group (Figure 6a). Transmission electron microscopy (TEM) showed increased mitochondrial density in these groups (Figure 6b). Immunohistochemical analysis further confirmed that UCP1 protein expression was markedly elevated in the Flox-MLE and Flox-SCFAs groups (Figure 6c,d). In contrast, no obvious changes were observed in the AKO-MLE or AKO-SCFAs groups (Figure 6a–d). These findings indicate that MLE and SCFAs activate BAT in an AMPKα1-dependent manner.

**Figure 7:**
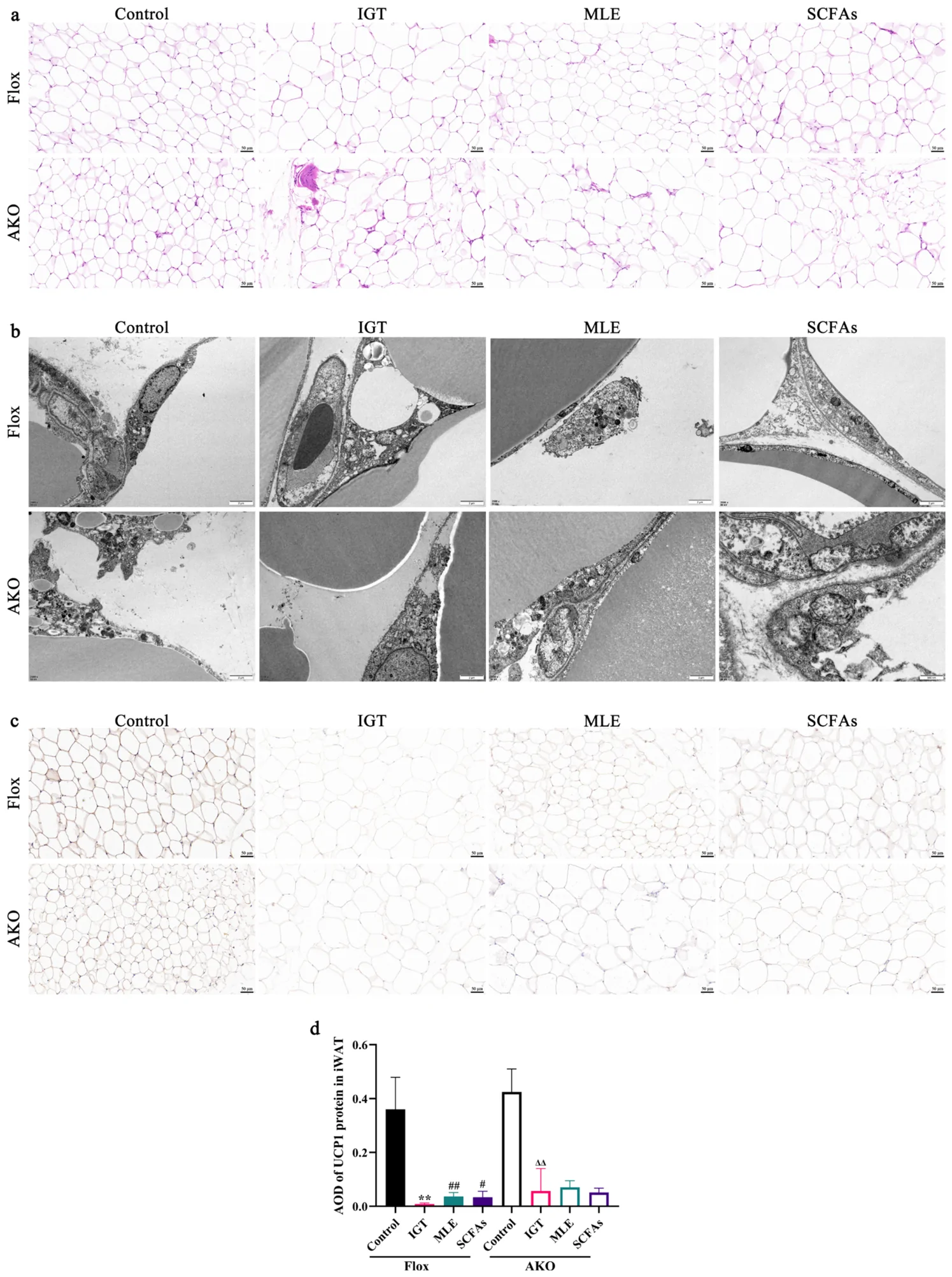
MLE and SCFAs improved iWAT architecture and promoted iWAT browning in IGT mice via AMPKα1. (**a**) H&E staining of iWAT (×20, scale bar = 50 μm). (**b**) Immunohistochemical staining of UCP1 in iWAT (×20, scale bar = 50 μm). (**c**) IWAT ultrastructure examined by TEM (×2500, scale bar = 2 μm). (**d**) Quantitative iWAT’ s UCP1 protein expression. Data are expressed as mean ± SD. *n* = 3. ** *p* < 0.01 vs. Flox-Control group. ^#^ *p* < 0.05, ^##^ *p* < 0.01 vs. Flox-IGT group. ^ΔΔ^ *p* < 0.01 vs. AKO-Control group.

In addition to BAT thermogenesis, WAT can undergo browning under certain conditions, such as cold exposure, giving rise to beige adipocytes that morphologically and functionally resemble brown adipocytes [16]. Histological examination of inguinal white adipose tissue (iWAT) using H&E staining, TEM, and immunohistochemistry indicated that in Flox mice, MLE or SCFAs intervention significantly ameliorated HFD-induced adipocyte disruption, increased mitochondrial density, and upregulated UCP1 protein expression (Figure 7a–d). However, no notable changes were detected in AKO mice receiving the same treatments (Figure 7a–d). Collectively, these results demonstrate that MLE and SCFAs promote WAT browning via AMPKα1.

### 2.6. MLE and SCFAs Reshaped Gut Microbiota and Increased SCFA Production in an AMPKα1-Dependent Manner

**Figure 8:**
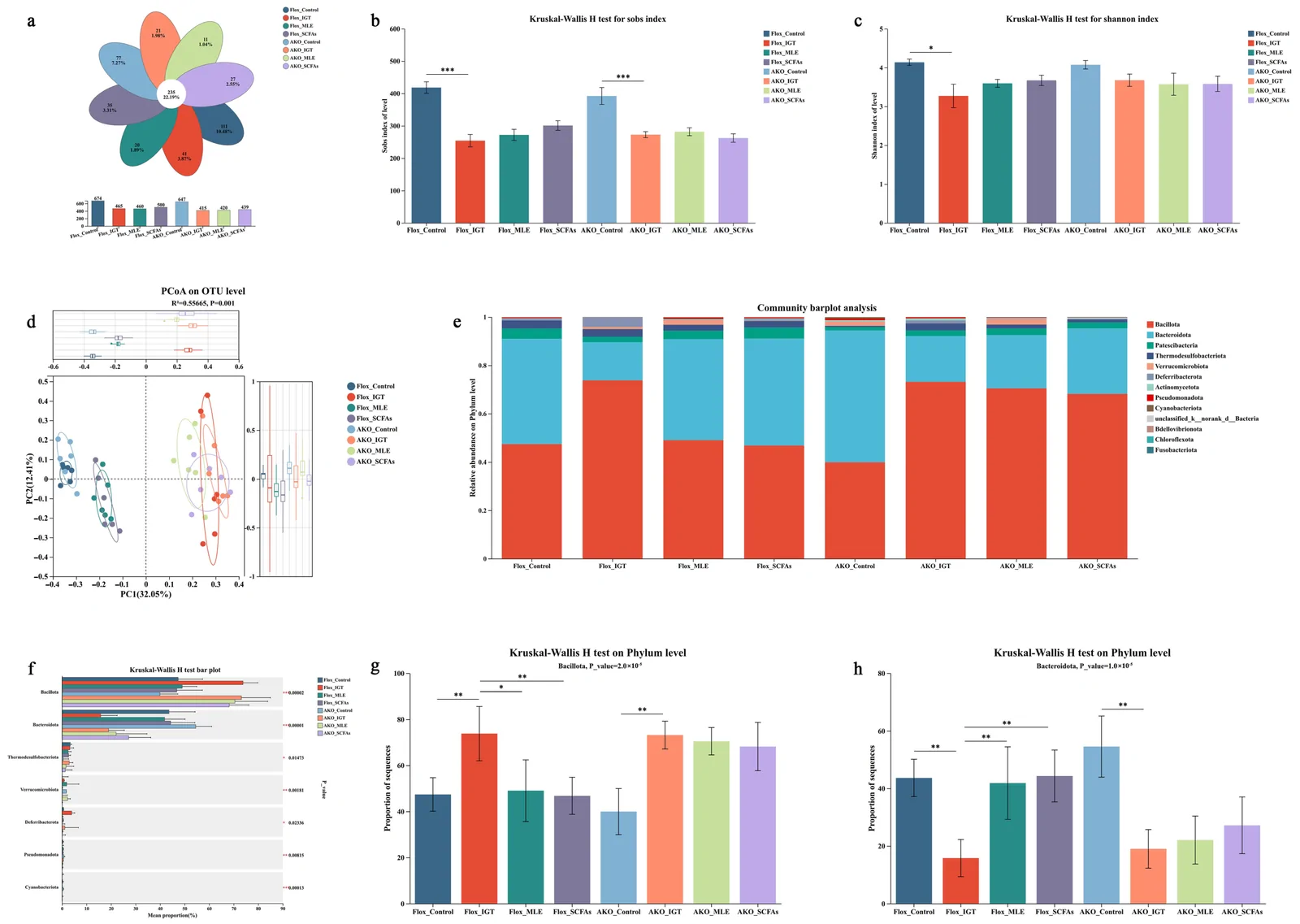
Gut microbiota assessment and phylum-level species analysis. (**a**) OTU Species Composition Venn diagram. (**b**) Sobs index. (**c**) Shannon index. (**d**) PCoA combined with Adonis tests. (**e**–**h**) Analysis of differential microbiota at the phylum level. Data are expressed as mean ± SD. *n* = 6. * *p* < 0.05, ** *p* < 0.01, *** *p* < 0.001.

To explore whether the metabolic benefits of MLE and SCFAs involve modulation of the gut microbiota, we performed 16S rRNA gene sequencing on fecal samples from 48 mice (6 per group). A total of 2,351,518 high-quality sequences were obtained. Species Venn diagram analysis revealed 235 operational taxonomic units (OTUs) shared across the eight groups, with the number of unique OTUs per group as follows: Flox-Control, 111; Flox-IGT, 41; Flox-MLE, 20; Flox-SCFAs, 35; AKO-Control, 77; AKO-IGT, 21; AKO-MLE, 11; and AKO-SCFAs, 27 (Figure 8a).

Alpha diversity analysis (Figure 8b,c) showed that Sobs and Shannon indices were significantly lower in the Flox-IGT group than in the Flox-Control group. Similarly, the AKO-IGT group had a significantly lower Sobs index relative to the AKO-Control group, while the Shannon index also decreased but without statistical significance. These results indicate a marked reduction in both species richness and diversity in IGT mice. Following the 10-week intervention, neither index changed significantly in any treatment group. However, the Flox-MLE and Flox-SCFAs groups showed an increasing trend relative to the Flox-IGT group, whereas no such trend was observed in the AKO treatment groups. Beta diversity assessed by principal coordinate analysis (PCoA) combined with Adonis tests revealed that (Figure 8d), compared with the Flox-IGT group, the fecal microbial community structure of the Flox-MLE and Flox-SCFAs groups shifted significantly toward that of the Flox-Control group. Conversely, the community compositions of the AKO-MLE and AKO-SCFAs groups remained similar to that of the AKO-IGT group.

Phylum-level analysis revealed that the gut microbiota in all groups consisted chiefly of *Bacillota*, *Bacteroidota*, *Patescibacteria*, *Thermodesulfobacteriota*, *Verrucomicrobiota*, *Deferribacterota*, *Actinomycetota*, *Pseudomonadota*, and *Cyanobacteriota*, with *Bacillota* and *Bacteroidota* being the most abundant (Figure 8e,f). Relative to their respective control groups, both the Flox-IGT and AKO-IGT groups exhibited a marked elevation in *Bacillota* abundance and a concurrent reduction in *Bacteroidota* abundance, leading to a markedly elevated *Bacillota*/*Bacteroidota* ratio (Figure 8g,h). After MLE or SCFAs treatment, the Flox mice displayed a significant reduction in *Bacillota* abundance, an elevation in *Bacteroidota*, and a subsequent decline in the *Bacillota*/*Bacteroidota* ratio. In AKO mice, only weak and non-significant trends toward similar changes were observed.

**Figure 9:**
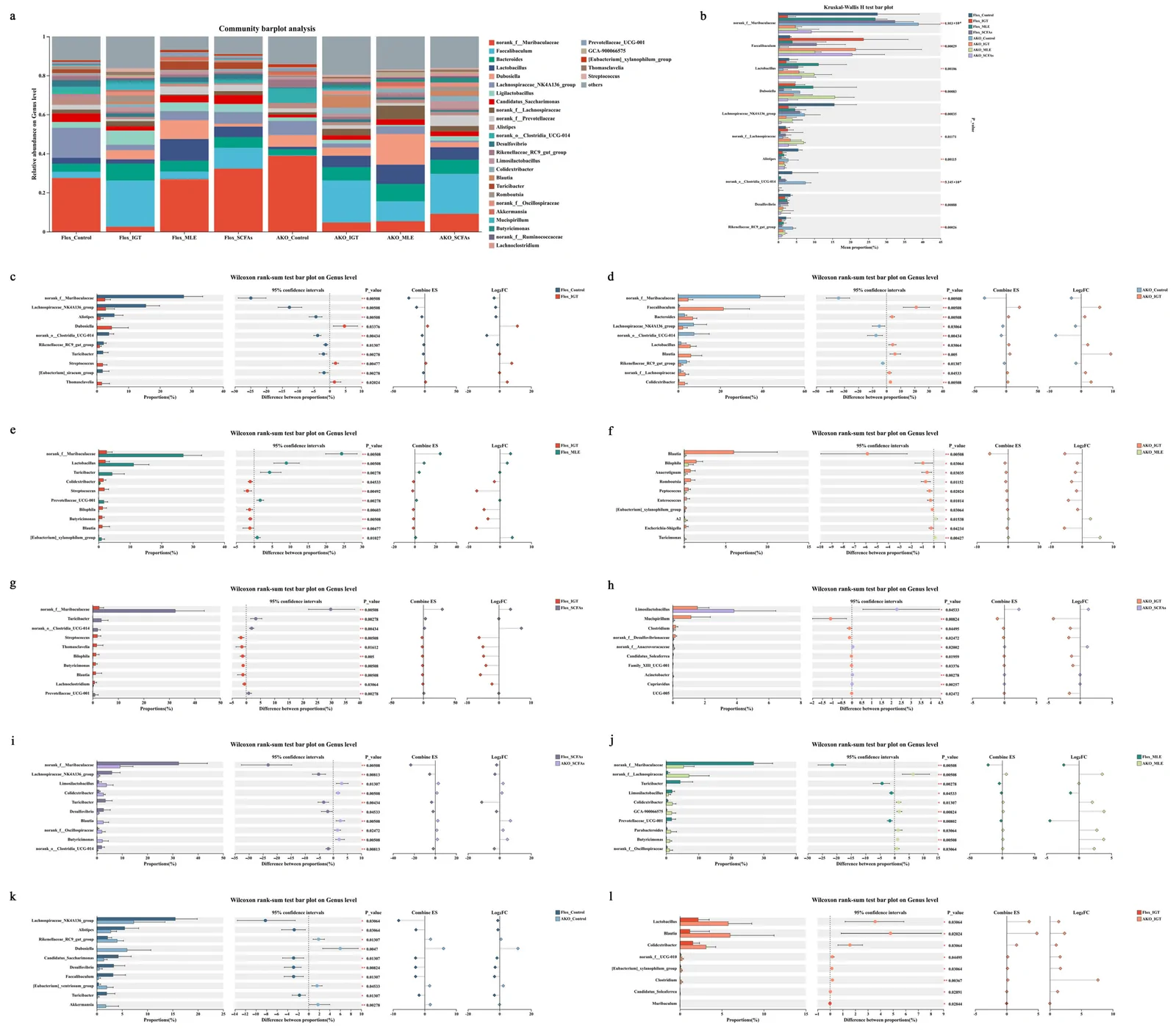
Genus-level species analysis. (**a**–**l**) Analysis of differential microbiota at the genus level. Data are expressed as mean ± SD. *n* = 6. * *p* < 0.05, ** *p* < 0.01, *** *p* < 0.001.

At the genus taxonomic level, a total of 30 predominant genera were recognized among the eight cohorts (Figure 9a,b). HFD-induced IGT caused gut dysbiosis in both Flox and AKO mice, characterized by a significant reduction in SCFAs-producing beneficial bacteria such as *norank_f__Muribaculaceae*, *Lachnospiraceae_NK4A136_group*, and *norank_o__Clostridia_UCG-014* (Figure 9c,d). Notably, the dysbiosis patterns differed between genotypes: the Flox-IGT group showed increased abundance of opportunistic pathogens like *Streptococcus* and *Thomasclavelia*, whereas the AKO-IGT group displayed elevated levels of *Blautia*, *Colidextribacter*, and other genera (Figure 9c,d). Following MLE or SCFAs treatment, the gut microbiota of Flox mice was effectively restored. Compared with the Flox-IGT group, the Flox-MLE and Flox-SCFAs groups were significantly enriched in beneficial bacteria such as *norank_f__Muribaculaceae*, *Turicibacter*, and *Prevotellaceae_UCG-001*, while the abundances of *Streptococcus* and *Bilophila* were markedly reduced (Figure 9e,g). In contrast, neither intervention fully restored the core microbiota in AKO mice. Specifically, relative to the AKO-IGT group, the AKO-MLE group decreased the abundance of *Blautia*, *Bilophila*, and *Escherichia-Shigella* while increasing *A2* and *Turicimonas* (Figure 9f). The AKO-SCFAs group downregulated harmful bacteria such as *Mucispirillum* and *norank_f__Desulfovibrionaceae* and enriched *Limosilactobacillus* (Figure 9h). However, neither treatment significantly increased the abundance of key SCFAs-producing genera such as *norank_f__Muribaculaceae*. Direct comparison between Flox and AKO intervention groups further underscored this difference (Figure 9i,j): the AKO-MLE and AKO-SCFAs groups had significantly lower abundances of *norank_f__Muribaculaceae* and *Turicibacter* than their Flox counterparts, while *Blautia*, *Butyricimonas*, and *Colidextribacter* remained more abundant in AKO mice. Additionally, at baseline, the AKO-Control group already exhibited reduced abundances of *Lachnospiraceae_NK4A136_group*, *Alistipes*, and *Turicibacter* compared to the Flox-Control group (Figure 9k). Under IGT conditions, the AKO-IGT group showed significantly lower *Muribaculum* abundance than the Flox-IGT group (Figure 9l).

**Figure 10:**
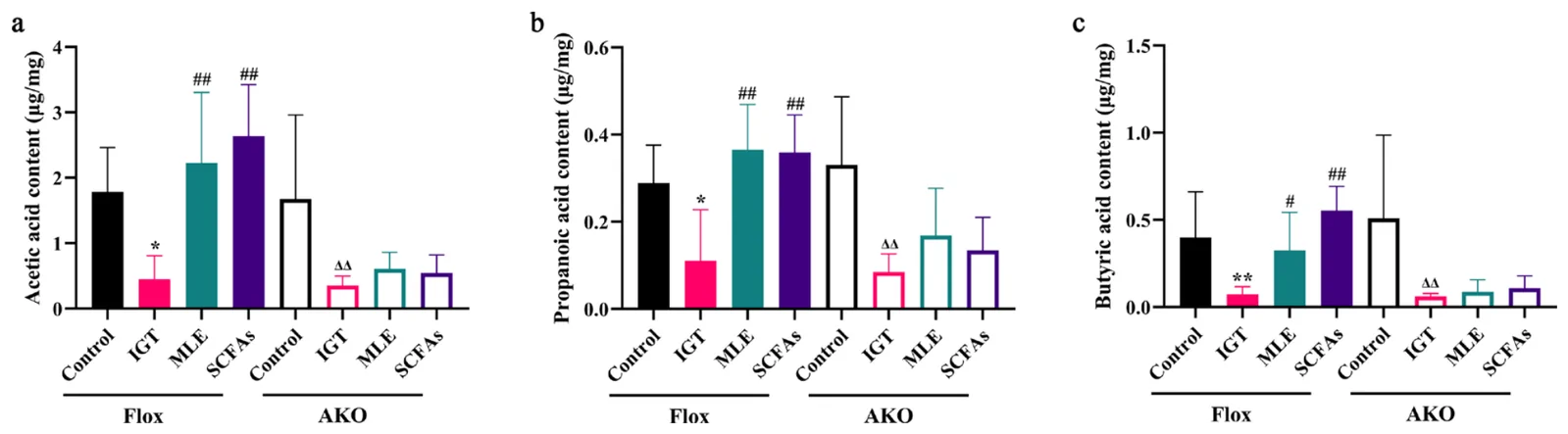
Quantification of fecal SCFAs. (**a**) Fecal acetic acid content. (**b**) Fecal propionic acid content. (**c**) Fecal butyric acid content. Data are expressed as mean ± SD. *n* = 6. * *p* < 0.05, ** *p* < 0.01 vs. Flox-Control group. ^#^ *p* < 0.05, ^##^ *p* < 0.01 vs. Flox-IGT group. ^ΔΔ^ *p* < 0.01 vs. AKO-Control group.

Fecal SCFA measurements revealed that (Figure 10a–c), relative to their respective Control groups, IGT groups had significantly reduced concentrations of acetic, propionic, and butyric acids. After 10 weeks of MLE or SCFAs treatment, Flox mice displayed significantly increased levels of all three SCFAs compared to the Flox-IGT group. In contrast, AKO mice receiving the same interventions showed only a non-significant trend toward elevation.

### 2.7. MLE and SCFAs Improved IGT via the AMPKα1/SIRT1/PGC-1α Pathway

**Figure 11:**
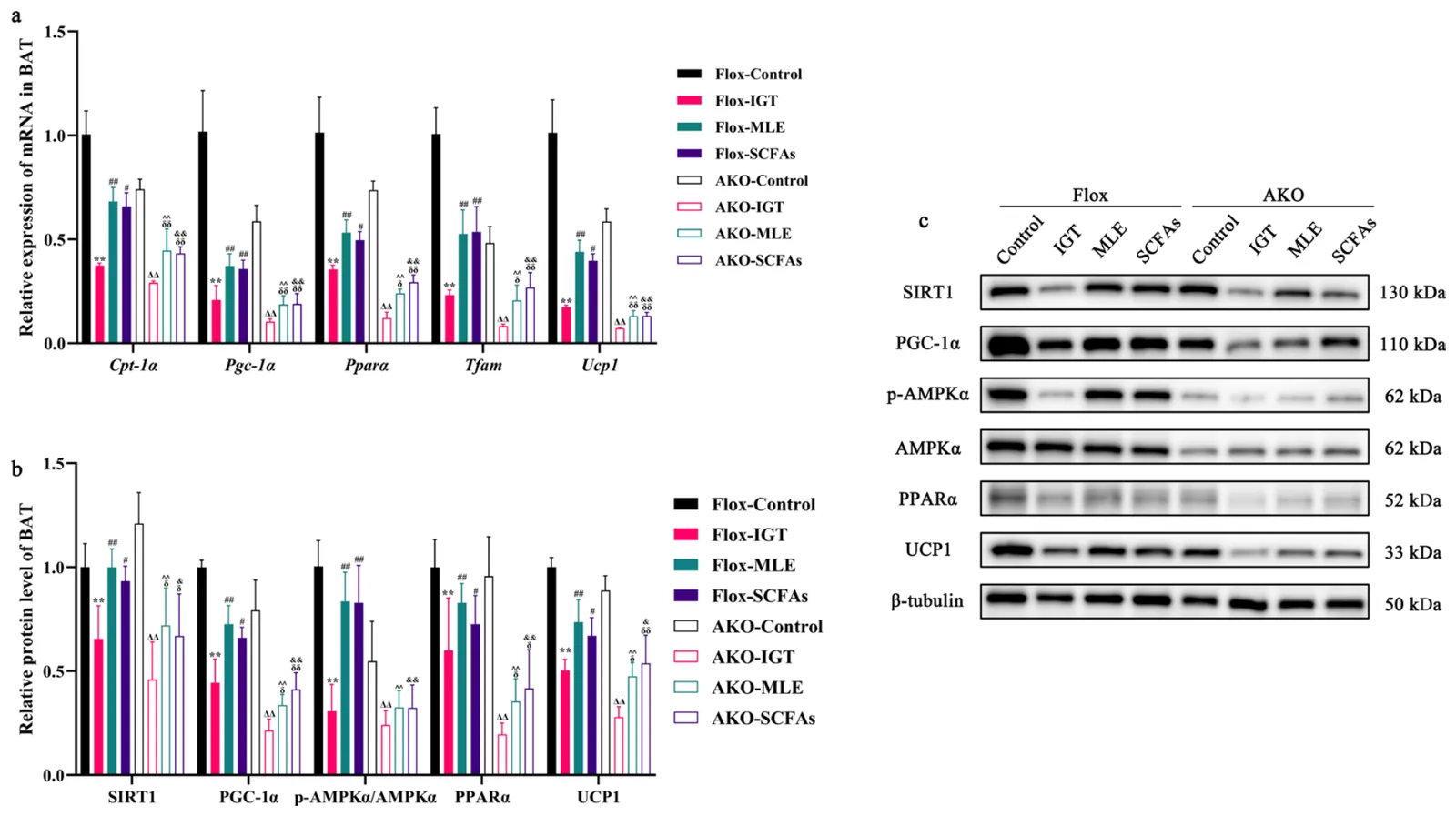
MLE and SCFAs promoted BAT activation through the AMPK/SIRT1/PGC-1α pathway. (**a**) Relative mRNA levels of genes expressed in BAT. (**b**) Relative protein expression of genes expressed in BAT. (**c**) Representative Western blot analysis of relative protein expression in BAT. Data are expressed as mean ± SD. *n* = 3. ** *p* < 0.01 vs. Flox-Control group. ^#^ *p* < 0.05, ^##^ *p* < 0.01 vs. Flox-IGT group.^^ *p* < 0.01 vs. Flox-MLE group. ^&^ *p* < 0.05, ^&&^ *p* < 0.01 vs. Flox-SCFAs group. ^ΔΔ^ *p* < 0.01 vs. AKO-Control group. ^δ^ *p* < 0.05, ^δδ^ *p* < 0.01 vs. AKO-IGT group.

**Figure 12:**
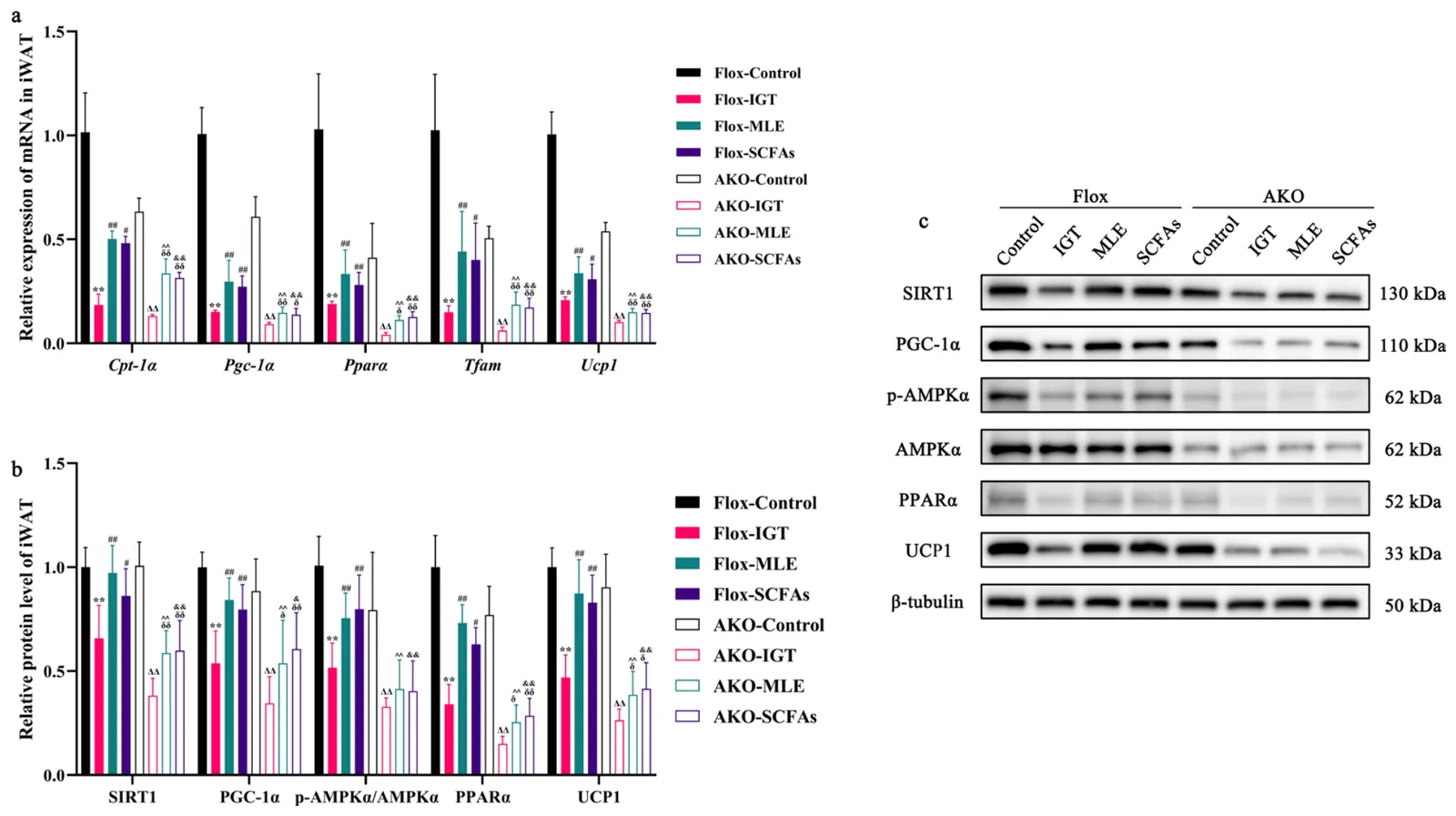
MLE and SCFAs promoted iWAT browning through the AMPK/SIRT1/PGC-1α pathway. (**a**) Relative mRNA levels of genes expressed in iWAT. (**b**) Relative protein expression of genes expressed in iWAT. (**c**) Representative Western blot analysis of relative protein expression in iWAT. Data are expressed as mean ± SD. *n* = 3. ** *p* < 0.01 vs. Flox-Control group. ^#^ *p* < 0.05, ^##^ *p* < 0.01 vs. Flox-IGT group. ^^ *p* < 0.01 vs. Flox-MLE group. ^&^ *p* < 0.05, ^&&^ *p* < 0.01 vs. Flox-SCFAs group. ^ΔΔ^ *p* < 0.01 vs. AKO-Control group. ^δ^ *p* < 0.05, ^δδ^ *p* < 0.01 vs. AKO-IGT group.

To further elucidate the molecular mechanisms by which MLE and SCFAs ameliorate IGT, we examined the expression of key genes and proteins involved in the AMPKα1/SIRT1/PGC-1α signaling pathway in BAT and iWAT using qRT-PCR and Western blotting. In Flox mice, treatment with MLE or SCFAs significantly upregulated the mRNA levels of BAT signature genes (*Ucp1*, *Pgc-1α*, and *Pparα*), the thermogenic factor *Cpt-1α*, and the mitochondrial regulator *Tfam* in both BAT (Figure 11a) and iWAT (Figure 12a). Consistently, Western blot analysis revealed a marked increase in the phosphorylated-AMP-activated protein kinase α (p-AMPKα)/total AMPKα ratio, as well as elevated protein expression of SIRT1, PGC-1α, peroxisome proliferator activated receptor α (PPARα), and UCP1 (Figure 11b,c and Figure 12b,c). In contrast, in AKO mice, p-AMPKα and total AMPKα proteins were nearly undetectable (Figure 11c and Figure 12c), confirming successful adipose-specific knockout of AMPKα1; the residual signal is attributable to the AMPKα2 isoform. Notably, in AKO mice, MLE and SCFAs treatment still led to a mild but significant upregulation of downstream genes and proteins compared with the AKO-IGT group (Figure 11b and Figure 12b). However, the magnitude of these increases was substantially lower than that observed in the corresponding Flox treatment groups (Figure 11b and Figure 12b). Collectively, these findings indicate that MLE and SCFAs act through the AMPKα1/SIRT1/PGC-1α axis to improve glucose intolerance, and that AMPKα1 is indispensable for the robust induction of thermogenic gene and protein expression in adipose tissue.

## 3. Discussion

In recent years, due to the influence of dietary patterns and lifestyle factors, the prevalence of IGT has continued to rise. According to the 11th edition of the IDF Diabetes Atlas (2025), approximately 635 million adults worldwide suffered from IGT [17]. Long-standing IGT can progress to T2DM and precipitate multiple complications [18]. Early effective intervention during the prediabetic stage is therefore a pivotal strategy not only to treat IGT but also to prevent T2DM [19].

Previous studies have elucidated several mechanisms underlying the glucose- and lipid-lowering effects of mulberry leaf, among which the gut microbiota and its metabolites, SCFAs, play a significant role. Mulberry leaf has been shown to increase gut microbial diversity and elevate SCFAs levels in T2DM mice [20]. Additionally, AMPK, a key regulator of bioenergetic metabolism, is essential for maintaining glucose homeostasis. Research indicates that MLE ameliorates T2DM by activating the AMPK/SIRT1/PGC-1α pathway, thereby promoting WAT browning and BAT activation [12]. However, it is unclear how the gut-adipose tissue axis mediates the glucose-lowering effect of mulberry leaf, especially in IGT. The crucial role of adipose AMPKα1 in the metabolic benefits of MLE and SCFAs has not been directly validated using genetic models. Most previous studies relied on AMPK activators or inhibitors, lacking tissue specificity; consequently, it remained unclear whether adipose AMPKα1 is indispensable for the anti-IGT effect of MLE or whether MLE activates adipose AMPKα1 via the gut microbiota/SCFAs axis. This study provides, for the first time, genetic evidence from tissue-specific knockout mice, demonstrating the essential role of adipose AMPKα1 in the ameliorative effect of mulberry leaf on IGT.

In the present study, we used AKO and Flox mice to investigate the role of adipose AMPKα1 in MLE-mediated amelioration of HFD-induced IGT. In Flox mice, MLE and SCFAs effectively reversed IGT-associated phenotypes: reducing body weight and fasting blood glucose, restoring glucose tolerance, lowering serum insulin and lipid levels, alleviating hepatic steatosis and inflammation, enhancing adaptive thermogenesis, increasing BAT activity, and promoting iWAT browning. In marked contrast, these effects were completely absent in AKO mice. These findings demonstrate that adipose AMPKα1 is critical for the systemic metabolic improvements conferred by MLE and SCFAs, consistent with the established role of AMPK as a central energy sensor integrating nutritional and hormonal signals to regulate energy balance [21]. In adipose tissue, AMPK activation promotes lipolysis [22], enhances glucose uptake [23], and boosts mitochondrial biogenesis [24], all of which contribute to improved insulin sensitivity and augmented thermogenesis.

A bidirectional crosstalk exists between the gut microbiota and host metabolism. Metabolic and inflammatory states modulate microbial community structure, and in turn, microbial composition influences systemic energy balance [25]. Our study provides multiple lines of evidence that the gut microbiota/SCFAs axis mediates the anti-IGT action of MLE. The *Bacillota*/*Bacteroidota* ratio is a key indicator of gut ecological balance and health, with an elevated *Bacillota*/*Bacteroidota* ratio being a well-recognized biomarker of obesity [26]. 16S rRNA sequencing revealed that in Flox mice, MLE treatment lowered the *Bacillota*/*Bacteroidota* ratio, enriched SCFAs-producing beneficial bacteria such as *norank_f__Muribaculaceae* and *Lachnospiraceae_NK4A136*, and reduced opportunistic pathogens such as *Streptococcus* and *Thomasclavelia* [27,28,29,30]. Moreover, MLE significantly increased fecal levels of acetic acid, propionic acid, and butyric acid in Flox mice, and these increases correlated positively with improvements in metabolic parameters. Notably, the phenotypic outcomes, along with gut microbiota and SCFA alterations, in the Flox-SCFAs group closely mirrored those observed in the Flox-MLE group. These results align with a growing body of literature showing that dietary polyphenols and polysaccharides from natural products, including mulberry leaf, exert prebiotic-like activity by promoting the growth of SCFAs-producing bacteria [12,31,32]. After absorption into the portal circulation, SCFAs can act directly on adipose tissue via GPR41 and GPR43, thereby activating AMPK and triggering downstream thermogenic effects [33,34].

An unexpected finding was that MLE-induced alterations in gut microbiota composition and increases in fecal SCFAs were almost completely abrogated in AKO mice. A possible explanation is that, owing to the lack of adipose AMPKα1, AKO mice fail to achieve systemic metabolic improvement and remain in a chronic IGT state, which persistently exerts detrimental effects on the gut microbiota. Additionally, factors such as ADP and FGF21 are known to influence intestinal barrier function and immune microenvironments via the circulation, thereby shaping the gut microbiota [35,36]. This mechanism is consistent with the observation that AKO mice exhibited lower serum ADP and FGF21 concentrations.

Central to the integration of cellular energy sensing and mitochondrial function is the AMPK/SIRT1/PGC-1α axis [37]. In this study, MLE and SCFAs significantly increased the mRNA levels of *Cpt-1α*, *Pgc-1α*, *Pparα*, *Tfam*, and *Ucp1* in both BAT and iWAT of Flox mice, promoted AMPKα phosphorylation, and increased the protein levels of SIRT1, PGC-1α, PPARα, and UCP1. AMPK and SIRT1 synergistically stimulate PGC-1α via phosphorylation and deacetylation, respectively, thereby promoting thermogenesis in BAT and iWAT [38,39,40]. MLE and SCFAs thus exert anti-IGT effects precisely by activating the AMPK pathway and promoting adipose thermogenesis.

Despite the lack of improvement in metabolic phenotypes and microbiota indices in AKO mice, we still detected a modest but significant upregulation of the relevant genes and proteins by qRT-PCR and western blot after MLE or SCFAs treatment, albeit far lower than in Flox mice. Potential explanations include the residual expression of AMPKα2 (the other α isoform) in AKO mice; however, AMPKα2 exhibits higher expression in liver and skeletal muscle, and its compensatory role in adipocytes remains controversial [41]. Other kinases, such as p38 MAPK, ERK, and PKA, are known to regulate PGC-1α and UCP1 expression independently of AMPK [42,43]. SCFAs can activate these kinases through G protein-coupled receptors, and flavonoids in MLE (e.g., quercetin) have been shown to stimulate p38 MAPK in adipocytes [44]. Nevertheless, it must be emphasized that this weak thermogenic activation did not translate into systemic metabolic benefits in AKO mice and was insufficient to drive overall energy expenditure. This further supports the primary role of adipose AMPKα1 in the anti-IGT effect of MLE.

Admittedly, direct microbiota transplantation or depletion experiments were not conducted in this study; therefore, causality requires further validation using germ-free mice or antibiotic-treated models. In addition, pharmacokinetic characterization of MLE and its major bioactive components was not performed in the present investigation, and such data would be valuable for future translational development. We plan to address these aspects in our subsequent studies. Overall, this study demonstrates that adipose AMPKα1 is an essential target for MLE in improving IGT and clarifies the mechanism by which MLE acts through the gut microbiota/SCFAs/AMPKα1 pathway. Our findings provide a scientific basis for the use of mulberry leaf as a prebiotic or functional food for the treatment of IGT and early-stage diabetes and lay a theoretical foundation for developing natural products or prebiotics that target adipose AMPKα1.

## 4. Materials and Methods

### 4.1. Drug Preparation

#### 4.1.1. Preparation of MLE

Mulberry leaves were obtained from Beijing Taiyang Shukang Pharmaceutical Co., Ltd. (2404023), Beijing, China. MLE was prepared following a previously established protocol from our group. First, 1 kg of mulberry leaves was soaked in 10 volumes (*v*/*w*) of deionized water for 30 min, boiled for 2 h, and then filtered. The residue was further decocted with 8 volumes (*v*/*w*) of deionized water for 2 h and filtered again. The combined filtrates were then concentrated by water bath and oven-dried under vacuum, affording 225.3 g of dried extract, equivalent to a 22.53% (*w*/*w*) yield. The dried extract was reconstituted with deionized water to a concentration equivalent to 0.4 g of raw material per milliliter, which corresponds to an in vivo dose of 4 g of dried mulberry leaves per kg of body weight—the optimal dose determined in our previous study [11]. The resulting solution was stored at −20 °C.

The chemical profile of MLE was previously characterized via UHPLC-Q-Orbitrap HRMS, with 1-deoxynojirimycin, chlorogenic acid, cryptochlorogenic acid, and rutin identified as its major bioactive constituents [12].

#### 4.1.2. Preparation of SCFAs

The sodium salts of acetate, propionate, and butyrate were dissolved in deionized water at a molar ratio of 67.5:25.9:40 to prepare SCFAs-supplemented drinking water, which was administered via the oral drinking route [12].

### 4.2. Animal Experiments

**Figure 13:**
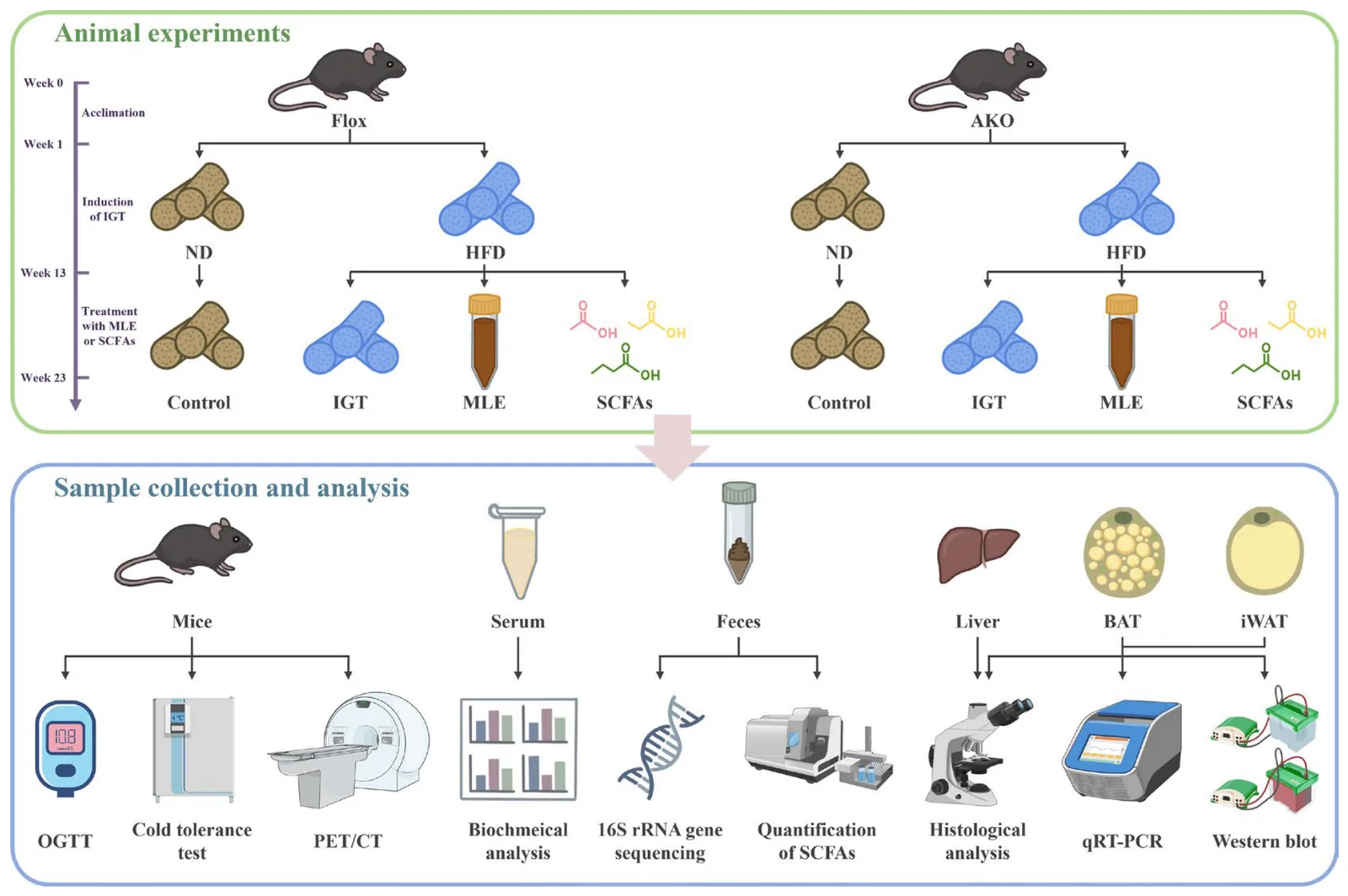
Experimental workflow.

All animal procedures were approved by the Animal Ethics Committee of Beijing University of Chinese Medicine (approval No. BUCM-2024082901-3120). Mice were housed in a barrier facility of the animal laboratory under controlled conditions (temperature 20–24 °C, relative humidity 50–70%, 12 h light/dark cycle) with free access to food and water. Animals were acclimatized for one week before the experiments, and the experimental process is shown in Figure 13.

To investigate whether MLE ameliorates IGT via AMPKα1 in adipose tissue, adipose tissue-specific AMPKα1 knockout (AKO) mice were generated by crossing Adipoq-Cre mice with AMPKα1-floxed (Flox) mice using CRISPR/Cas-mediated genome editing. Specific pathogen-free (SPF)-grade, 5-week-old male AKO mice (AMPKα1^fl/fl^, Adipoq-Cre) and their male littermate Flox control mice (AMPKα1^fl/fl^, Cre-negative), both on a C57BL/6JCya background, were obtained from Cyagen Bioscience Co. [SCXK (JI) 2021-003]. SPF-grade animals are defined as those that, in addition to the pathogens excluded from clean-grade animals, do not carry major potential infectious agents, opportunistic pathogens, or pathogens that could significantly interfere with scientific experiments.

Subsequently, therapeutic administration was initiated. The two HFD-fed groups were further randomized into the IGT model group, MLE-treated group, and SCFAs-treated group, and continued feeding the HFD. The two ND groups were fed ND as the control group. MLE was administered by oral gavage once daily at a dose equivalent to 4 g of dried mulberry leaves per kg of body weight, with a fixed volume of 0.1 mL per 10 g of body weight. SCFAs were dissolved in drinking water and provided ad libitum throughout the treatment period. Treatments lasted for 10 weeks, and OGTTs were repeated at weeks 9 and 10. Upon completion of treatment, fresh feces were collected for 16S rRNA gene amplicon sequencing and SCFAs quantification. Blood samples were obtained from the retro-orbital sinus for biochemical analyses. Liver, iWAT, and BAT were harvested, immediately frozen in liquid nitrogen, and stored at −80 °C for subsequent gene and protein expression assays.

### 4.3. OGTT

OGTT was performed at the 9th and 10th week of treatment. After fasting for 6 h, mice were orally administered glucose (2 g/kg). Blood glucose was monitored with a glucometer at time points of 0 (baseline), 15, 30, 60, 90, and 120 min following glucose loading. The ACCU-CHEK^®^ Performa blood glucose meter used in this study is manufactured by Roche Diagnostics, Indianapolis, IN, USA.

### 4.4. Cold Tolerance Test

A cold tolerance test was performed at the 10th week of treatment. Mice were housed in a temperature-controlled chamber at 4 °C for 4 h under fasting conditions with free access to water. Rectal temperature was measured at baseline (0 h) and at 1, 2, 3, and 4 h after cold exposure.

### 4.5. BAT PET/CT Imaging

At week 10 of treatment, BAT PET/CT imaging was conducted. Mice received [^18^F]-FDG (0.1 mL, 6.66–7.40 MBq) via tail vein injection. Isoflurane (4%) was used to induce anesthesia after 50 min, and the animals were placed in a prone position on the scanner bed. A two-bed CT acquisition was performed first with the following parameters: projections 180, binning 4 × 4, transaxial field of view 53.9 mm, axial scanning length 134 mm, tube voltage 80 kV, tube current 500 μA. Subsequently, PET imaging was conducted on the same scanner for 10 min. Data were processed using Inveon Research Workplace software (v4.2), which automatically calculated the standardized uptake value (SUV).

### 4.6. Biochemical Analysis

Serum levels of TC, TG, LDL-C, HDL-C, AST, and ALT were determined using commercial biochemical assay kits. Serum concentrations of INS, ADP, FFA, IL-6, IL-1β, FGF21, and TNF-α were measured via enzyme-linked immunosorbent assay (ELISA) kits.

### 4.7. H&E Staining

Liver, BAT, and iWAT were fixed in 4% paraformaldehyde, then dehydrated and embedded in paraffin. Sections were cut at a thickness of 4 μm, and subsequently stained with hematoxylin for 1–2 min and eosin for 2–3 min. The stained sections were examined under a Nikon Eclipse ci microscope (Nikon Corporation, Yokohama, Japan), and images were captured using a Nikon DS-U3 imaging system for analysis.

### 4.8. Oil Red O Staining

Frozen sections were rehydrated, air-dried, and fixed for 15 min, then stained in the dark with oil red O solution for 8–0 min. Subsequently, the sections were subjected to hematoxylin counterstaining for 3–5 min and treated with a bluing solution. Following glycerol gelatin mounting, micrographs were acquired and quantified with Image-Pro Plus 6.0 software.

### 4.9. Immunohistochemical Staining

Immunohistochemical staining was used to examine UCP1 expression in iWAT and BAT. Tissue paraffin sections were first dewaxed and rehydrated, then exposed to antigen retrieval, and finally treated to suppress endogenous peroxidase. Sections were exposed to a rabbit anti-UCP1 primary antibody (1:300, B112174, Servicebio, Wuhan, China) overnight at 4 °C, then incubated with HRP-conjugated goat anti-rabbit secondary antibody for 50 min at ambient temperature. DAB chromogenic reaction and hematoxylin counterstaining were performed, followed by dehydration and mounting of the sections. Micrographs were acquired and quantified with Image-Pro Plus 6.0 software.

### 4.10. TEM

Fresh BAT and iWAT tissues were immediately immersed in electron microscopy fixative and fixed at 4 °C for 2–4 h. Following a 2-h post-fixation in 1% osmium tetroxide/PBS at ambient temperature, the specimens were dehydrated via an ethanol gradient and infiltrated with resin. Ultrathin sections (60–80 nm) were prepared, double-stained with 2% uranyl acetate and lead citrate, and then viewed under a transmission electron microscope for mitochondrial morphology assessment.

### 4.11. 16S rRNA Gene Sequencing of Gut Microbiota

PCR amplification of the 16S rRNA V3–V4 region was performed using primers 338F (5′-ACTCCTACGGGAGGCAGCAG-3′) and 806R (5′-GGACTACHVGGGTWTCTAAT-3′), with fecal DNA extracted from mice as the template. Libraries were constructed with the NEXTflex^®^ Rapid DNA Sequencing Kit (Bioo Scientific Corporation, Austin, TX, USA) and sequenced on an Illumina NextSeq 2000 PE300 platform (Illumina, Inc., San Diego, CA, USA).

Raw paired-end reads were filtered using fastp (v0.23.4) and merged via FLASH (v1.2.11). The filtered and merged sequences were then assigned to OTUs at 97% similarity using USEARCH (v11), with chimeras eliminated. OTU taxonomy was determined with the RDP Classifier (v2.11) based on the Silva 138.2 database (70% confidence cutoff).

The Majorbio Cloud Platform (https://cloud.majorbio.com) was used for data analysis. Mothur (v1.30.2) was employed to compute alpha diversity indices and beta diversity metrics, with visualization performed in R (v3.3.1). Overall group differences in bacterial community composition were evaluated by PCoA (Bray-Curtis) combined with the Adonis test. Dominant taxa were shown using bar plots and heatmaps. For intergroup comparisons, the Kruskal-Wallis test (multiple groups) or Wilcoxon test (two groups) was applied in R.

### 4.12. Quantification of SCFAs

A 20-mg fecal aliquot was combined with 800 μL of 0.5% phosphoric acid containing the internal standard (2-ethylbutyric acid, 10 μg/mL). The mixture was cryogenically ground (50 Hz, 3 min), ultrasonicated for 10 min, and then spun at 13,000 rcf for 15 min at 4 °C. The supernatant was extracted with 200 μL of n-butanol by vortexing for 10 s and low-temperature ultrasonication for 10 min, followed by centrifugation at 13,000 rcf for 5 min at 4 °C. The supernatant obtained was subjected to gas chromatography-mass spectrometry (GC-MS) analysis on an HP FFAP capillary column (30 m × 0.25 mm × 0.25 μm). The carrier gas was high-purity helium (≥99.999%), and the injection parameters were set as 1 μL with a split ratio of 10:1. The column temperature was programmed as follows: initial 80 °C, ramped to 120 °C at 20 °C/min, then to 160 °C at 5 °C/min, with a post-run hold at 220 °C for 3 min. Electron impact ionization was used for mass spectrometry, with the ion source at 230 °C, the quadrupole at 150 °C, the transfer line at 230 °C, and the electron energy set to 70 eV. Selected ion monitoring mode was used for data acquisition. Target SCFA ion peaks were auto-integrated with the default settings of MassHunter quantitative software (v10.0.707.0) and subsequently verified manually. Concentrations of analytes in each sample were calculated from standard curves and converted to the actual content of SCFAs in the feces.

### 4.13. qRT-PCR Analysis

RNA extraction from total samples was accomplished using the RNAeasy™ Animal RNA Isolation Kit (Beyotime, Shanghai, China). RNA purity and concentration were assessed using a NanoDrop ONE (Thermo Fisher Scientific, Waltham, MA, USA); only samples with concentrations > 100 ng/µL, A260/A280 ratios between 1.8 and 2.2, and A260/A230 ratios > 2.0 were used for subsequent qRT-PCR analysis. cDNA was synthesized with HiScript III RT SuperMix (Vazyme, Nanjing, China), diluted, and then subjected to qPCR in duplicate using ChamQ SYBR Master Mix (Vazyme, Nanjing, China) on a QIAquant 96 system (QIAGEN, Hilden, Germany). Target gene expression was normalized against β-actin and analyzed with the 2^−ΔΔCt^ approach. All primer details are compiled in Table 1.

### 4.14. Western Blot Analysis

Protein samples (20 μg) extracted from BAT or iWAT were resolved on 10% SDS-PAGE gels and electroblotted onto PVDF membranes. The blots were treated with a protein-free rapid blocking buffer for 40 min and then incubated with primary antibodies for 12 h at 4 °C. The primary antibodies used were: p-AMPKα (1:1000, ab133448, Abcam, Cambridge, UK), AMPKα (1:1000, ab207442, Abcam), SIRT1 (1:1000, ab189494, Abcam), UCP1 (1:5000, ab209483, Abcam), PGC-1α (1:1000, ab313559, Abcam), PPARα (1 μg/mL, ab126285, Abcam), and β-tubulin (1:1000, ab179513, Abcam). Following three 10-min TBST washes, the blots were probed with HRP-conjugated goat anti-rabbit secondary antibody (1:5000, ab205718, Abcam) for 1 h at room temperature with gentle agitation, then subjected to three further 10-min TBST washes. Chemiluminescence was used for protein band detection, and signal quantification was carried out with ImageJ software (v1.54p).

### 4.15. Statistical Analysis

SPSS 27.0 and GraphPad Prism 10.1 were used for statistical evaluation. Results were presented as mean ± standard deviation (SD). Following normality assessment, group differences were analyzed by one-way analysis of variance (ANOVA) or nonparametric tests, with post-hoc least significant difference (LSD) testing for homogeneous variance data. Significance threshold was defined as *p* < 0.05.

## 5. Conclusions

In conclusion, this study provides the first genetic evidence that adipose AMPKα1 is essential for MLE to ameliorate high-fat diet-induced IGT. Using AKO mice, we demonstrated that MLE-induced improvements in glucose tolerance, insulin sensitivity, lipid profiles, hepatic steatosis, and systemic inflammation were completely abrogated in the absence of adipose AMPKα1. Mechanistically, MLE activated the AMPKα1/SIRT1/PGC-1α signaling pathway, promoting brown adipose tissue activation and inguinal white adipose tissue browning, which together enhanced adaptive thermogenesis. Concurrently, MLE reshaped gut microbiota composition—enriching SCFAs-producing bacteria and elevating fecal acetate, propionate, and butyrate levels. Notably, microbiota and SCFA changes were also absent in AKO mice, suggesting that gut microbiota remodeling is secondary to systemic metabolic improvement driven by adipose AMPKα1 activation, rather than a direct effect of MLE on the gut. Our findings identify adipose AMPKα1 as a critical molecular target for dietary interventions aimed at improving glucose metabolism in prediabetes. Future studies employing fecal microbiota transplantation or antibiotic-treated models are warranted to establish causality between gut microbiota changes and metabolic improvements. Overall, this work advances the understanding of the adipose–gut axis and supports the potential of mulberry leaf as a dietary strategy for prediabetes management.

## Figures and Tables

**Table 1:** Sequences of primers for qRT-PCR.

Primer	Forward	Reverse
*Ucp1*	GAAGGGACGCTCACCTTTGA	TAGGGGTCGTCCCTTTCCAA
*Pparα*	CCAGCAACAACCCGCCTTTT	GGAGAGCCCGCATTACCTAC
*Pgc-1α*	TTGACCCTGCTTCCACCAAG	GTCGCCCTTGTTCGTTCTGT
*Cpt-1α*	ATCAAGAAGTGCCGGACGAG	GAACCTCTGCTCTGCCGTTG
*Tfam*	CTCTTAGCGGTCCTGTCCAC	GAGCCGAATCATCCTTTGCC
*β-actin*	GCTACAGCTTCACCACCACA	AGGAAGGCTGGAAAAGAGCC

## Data Availability

The original contributions presented in this study are included in the article. Further inquiries can be directed to the corresponding author.

## Abbreviations

The following abbreviations are used in this manuscript:

AMPKα1	adenosine 5′-monophosphate-activated protein kinase α1
ADP	adiponectin
AKO	adipose tissue-specific AMPKα1 knockout
ALT	alanine aminotransferase
AUC	area under the curve
AST	aspartate aminotransferase
BAT	brown adipose tissue
ELISA	enzyme-linked immunosorbent assay
FGF21	fibroblast growth factor 21
FFA	free fatty acids
GC-MS	gas chromatography-mass spectrometry
H&E	hematoxylin and eosin
HDL-C	high-density lipoprotein cholesterol
HFD	high-fat diet
IGT	impaired glucose tolerance
iWAT	inguinal white adipose tissue
INS	insulin
IL-1β	interleukin-1β
IL-6	interleukin-6
LSD	least Significant Difference
LDL-C	low-density lipoprotein cholesterol
MLE	mulberry leaf water extract
ND	normal chow diet
ANOVA	one-way analysis of variance
OTUs	operational taxonomic units
OGTT	oral glucose tolerance test
OGTTs	oral glucose tolerance tests
PPARα	peroxisome proliferator activated receptor α
PGC-1α	peroxisome proliferator-activated receptor γ coactivator 1α
p-AMPKα	phosphorylated-AMP-activated protein kinase α
PCoA	principal coordinate analysis
SCFAs	short-chain fatty acids
SIRT1	sirtuin 1
SD	standard deviation
SPF	specific pathogen free
SUV	standardized uptake value
TC	total cholesterol
TEM	transmission electron microscopy
TG	triglycerides
TNF-α	tumor necrosis factor-α
T2DM	type 2 diabetes mellitus
UCP1	uncoupling protein 1
WAT	white adipose tissue
